# Spiritual Dimensions of Moral Injury: Contributions of Mental Health Chaplains in the Canadian Armed Forces

**DOI:** 10.3389/fpsyt.2018.00592

**Published:** 2018-11-14

**Authors:** Lorraine A. Smith-MacDonald, Jean-Sébastien Morin, Suzette Brémault-Phillips

**Affiliations:** ^1^Faculty of Nursing, University of Calgary, Calgary, AB, Canada; ^2^Royal Canadian Chaplain Service (RCChS), Ottawa, ON, Canada; ^3^Department of Occupational Therapy, Faculty of Rehabilitation Medicine, University of Alberta, Edmonton, AB, Canada

**Keywords:** moral injury, chaplains, mental health, intervention, spirituality, interdisciplinary collaboration

## Abstract

Moral Injury (MI) describes the profound distress experienced by military personnel as a result of a violation of personal beliefs. Impacting not only psychological, but spiritual, health, and well-being, MI is associated with spiritual/religious (S/R) suffering and a need to find hope, trust, connection, reconciliation, and wholeness. Addressing spiritual wounds can help military personnel overcome fundamental barriers that may impede them from effectively engaging in or benefitting from traditional trauma therapies and having a more complete recovery. Military Chaplains in the Canadian Armed Forces (CAF) are both embedded with the troops in garrison and theater and work closely with service providers such as the Royal Canadian Medical Services. In their role, they offer front-line support and services to members and their families and facilitate access to care. Specific to the assessment and treatment of MI, Mental Health Chaplains (MHCs) offer S/R expertise and a complimentary clinical skill set to service members and interdisciplinary teams. This perspectives article explores the S/R dimension of MI, discusses the role of MHCs in CAF Mental Health (MH) Clinics, and provides clinical perspectives of a MHC regarding the treatment of MI. Key focuses of MHC interventions include bridging to other mental health services and supports, facilitating S/R coping and grounding, reconciling worldviews, resolving anger at a God-figure (not specific to any S/R perspective) and fostering reconciliation. Based on the literature, Mental Health practitioner's feedback, and clinical experience, MHCs are integral to service provision regarding MI and warrant more widespread inclusion on interdisciplinary teams in CAF MH Clinics.

## Introduction

Interest in military and veteran health and well-being has recently been reignited by conflicts involving urban guerrilla warfare. During both combat and peace-keeping missions, military personnel can be exposed to morally injurious experiences (MIEs) in which they face ethical dilemmas. Soldiers involved in the wars in Afghanistan and Iraq reported MIEs such as firing at the enemy (52%), being responsible for killing (40%) including the death of a non-combatant (20%), witnessing and being unable to help ill/wounded women and children (60%), and facing ambiguous ethical situations (27%) (1). Intense and overlapping emotional, cognitive, and spiritual/religious (S/R) distress can occur as a result of MIEs, and manifest as depression, anxiety, post-traumatic stress disorder (PTSD) and moral injury (MI) (2–5).

MI is described by Litz et al. as “perpetrating, failing to prevent, bearing witness to, or learning about acts that transgress deeply held moral beliefs” (5). Associated with feelings of guilt, shame, anxiety, and anger, MI can have a profound and enduring impact. Spiritually, it can result in the shattering of a person's core sense of self, connection with self, others and the sacred or Transcendent, and a loss of meaning, purpose, trust, and hope (3, 6, 7). Morally injurious transgressions against self and others have been identified as the most significant factors associated with suicidal ideation in veterans (8, 9). Further, MI is strongly and independently associated as a risk factor for suicide among veterans and active duty military personnel with PTSD symptoms (3, 8, 10).

This manuscript describes the importance of addressing the spiritual dimension of MI, explores the contributions of Mental Health Chaplains (MHCs) on interdisciplinary (ID) healthcare teams in the Canadian Armed Forces (CAF), and advocates for their more widespread inclusion in the treatment of MI.

## Spirituality and moral injury

Spirituality and spiritual distress have been recognized in the literature as core features of MI (11, 12), with some scholars considering MI to be a form of S/R struggle (13–16). Studies examining first-hand experiences of military personnel found that S/R struggles and existential crises were commonly reported following exposure to MIEs (17–19). MIEs and MI appear to challenge a person's spirituality, sense of self and spirit, underlying core beliefs, and fundamental relationships with self, others and the sacred/Transcendent (14, 17, 20–22). Due to commonly held North American morals, beliefs, and values (which inherently have S/R underpinnings), spiritual distress can be experienced by individuals who do or do no identify with a S/R perspective.

S/R has been found to be positively related to an individual's ability to integrate stressful life events into their personal framework and experiences (23). Factors that are independently linked to post-traumatic growth (PTG) and increased well-being among veterans include intrinsic religiosity, spirituality, and purpose in life (24, 25). It has been suggested that S/R beliefs often enable individuals to engage with existential questions or those related to meaning and purpose (26). Hijazi et al. (25) argues that a transformation from struggle to PTG can occur because “deeply held beliefs spark a question for re-establishing meaning, reformulating shattered beliefs about goodness and one's worth, and seeking forgiveness from self and others, which is what may ultimately facilitate growth” (25). While S/R struggles are likely to be initially associated with lower well-being, individuals who are able to find a sense of meaning or spiritual significance in their struggles have been shown to have higher levels of well-being and lower levels of depression and anxiety (27).

Conversely, while S/R may facilitate PTG and well-being, one's S/R framework may also increase the risk of MIEs and MI. Ames et al. (3) found that religiosity does not mediate nor moderate the relationship between MI and suicide among veterans/active duty military with PTSD symptoms (3). Military personnel who adhere to strict religious principles and have high moral expectations can potentially experience heightened feelings of guilt and self-condemnation following a MIE, further increasing their risk of MI (28, 29). Hufford et al. (20) suggest that this may be due to existential questioning of a Divine being and “shattering [of] deeply held spiritual beliefs” (20). Incongruence to one's S/R framework or belief system may create intense experiences of distress, especially regarding one's relationship with a higher power (30). In response to the loss of meaning and S/R distress, some individuals seek opportunities that can help restore meaning and relationships through previously established S/R frameworks.

Spiritual struggles and illnesses (31–34) often reside within and mimic mental health concerns developed during military service. While addressing S/R can help to disentangle underlying MI causes, particularly as they relate to S/R struggles and illnesses, many healthcare providers are either unaware of how to approach this domain or uncomfortable doing so. Studies highlight that many mental health professionals have received little to no training in and have limited knowledge about what to do with S/R aspects of service provision. As a result, while a limited number of clinicians address S/R within the therapeutic context (35), many more tend to neglect it in practice (36). In addition, assessments tend to overlook S/R dimensions of service members. Impediments to the inclusion of S/R in mental health treatment may also relate to: (1) therapist biases, (2) scientific avoidance, skepticism, or antagonism toward S/R, or (3) illiteracy regarding S/R perspectives, processes and practices (37–39). These barriers may result in the S/R dimension of MI being overlooked, and a potentially essential, if not foundational aspect of healing, being negated or untapped.

## Mental health chaplains in the canadian armed forces

The CAF has a long-standing tradition of employing Chaplains to provide S/R leadership and support. Military Chaplains are both embedded with the troops in garrison and theater and work closely with various care providers such as the Royal Canadian Medical Services (RCMS). In so doing, they offer front-line support and services to members and their families and facilitate access to care. Most recently, the Royal Canadian Chaplain Service (RCChS) was commended for its contributions during operations in Afghanistan (2001–2010). The establishment of MHC roles within CAF Mental Health (MH) Clinics has evolved from these collaborations and in response to the needs of military members experiencing spiritual and mental health concerns such as operational stress injuries (OSIs) including PTSD and MI. Colleagues and service members alike have attested to their role and contribution.

MHCs, who are first and foremost Chaplains with a primary call to serve members and their families, are also trained as counselors or psychotherapists. Enriched by this dual training, MHCs provide a specialized counseling ministry centered on S/R struggles and distress. Drawing on both spiritual and counseling modalities and practices, MHCs approach service members using a person-centered, holistic approach that sees each member as a *human being*—with a body, mind, spirit—who relies on connection and a sense of belonging to thrive. From the MHC perspective, the human “spirit” is understood to be “the essential core of the individual – the deepest part of the self” which is thoroughly manifested in behavioral, relational, and vocational choices and personal identity (40).

**Figure 1:**
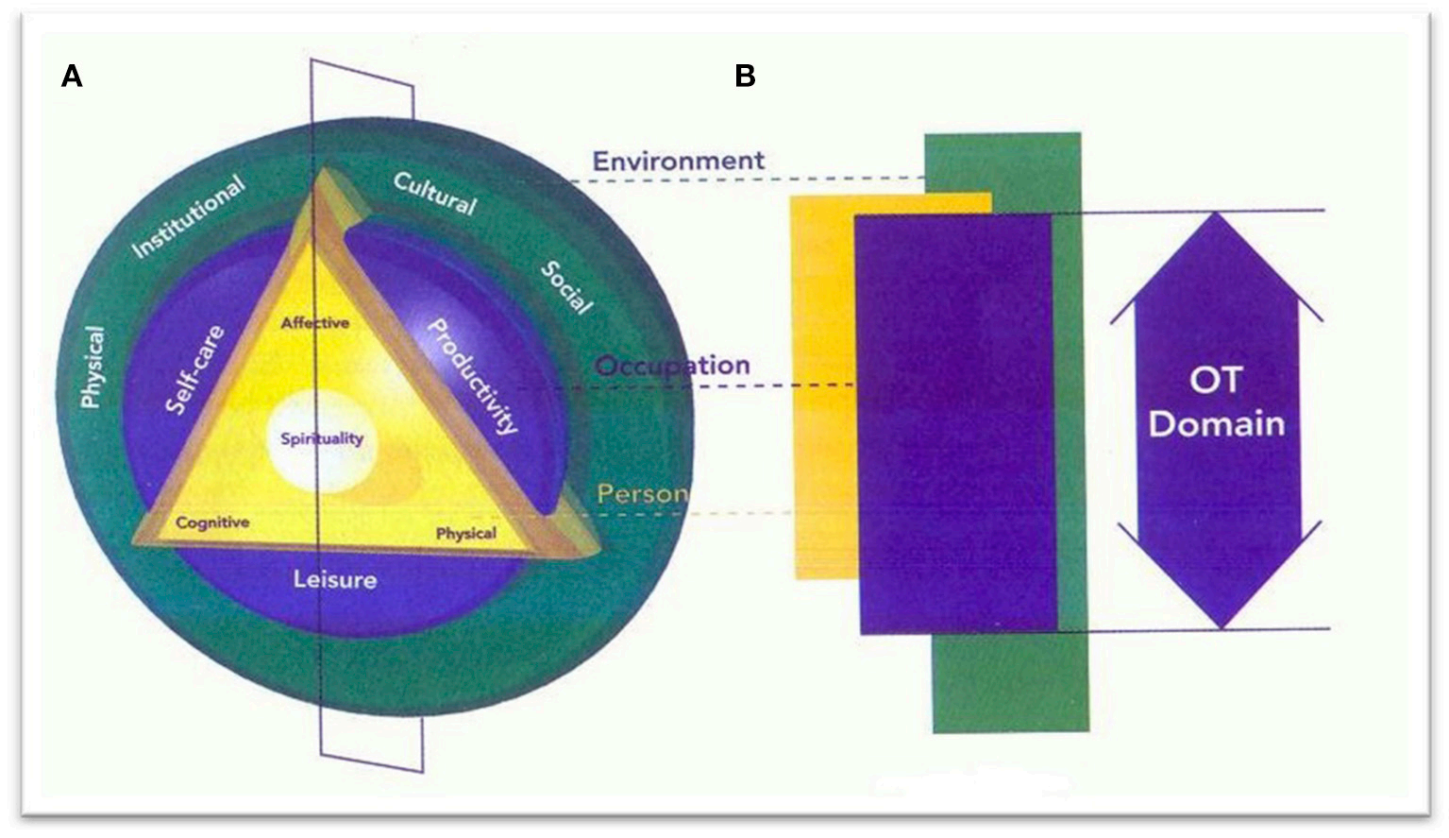
Canadian model of occupational performance and engagement (CMOP-E).

**Figure 2:**
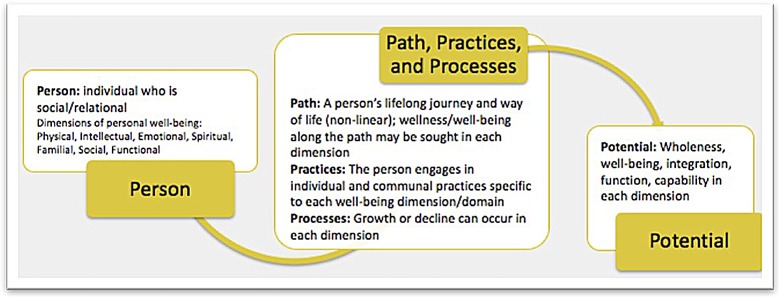
The 5 Ps: personal paths, practices and processes to maximizing potential.

To holistically support military personnel, MHCs employ a biopsychosocial-spiritual (BPSS) model such as the Canadian Model of Occupational Performance and Engagement (Figure 1) (41) that views the biological, psychological, social, and spiritual dimensions as distinct, yet interconnected and inseparable (42). With an emphasis on the spiritual domain, MHCs are predominantly focused on S/R processes (e.g., struggles, questions, wounds), barriers that may delimit or impede overall success, as well as S/R resources and practices that can facilitate recovery and resilience. MHCs also use S/R practices (e.g., prayer, meditation, rituals), explore issues of meaning and purpose, work through S/R and existential questions, address fractured worldviews, core beliefs, and relationships, and facilitate movement toward recovery, reconciliation, and restoration (43). Addressing the spiritual domain in this way not only helps to address and heal specific spiritual wounds, but encourages service members to engage in healthy S/R practices and processes that enable them to reach their personal potential (see Figure 2) [Brémault-Phillips et al., unpublished; (44)].

In addition to addressing S/R topics that may arise during treatment for MI, MHCs also provide personal and professional support to healthcare providers. Regarding service provision to military personnel, MHCs facilitate integration of S/R-related components into care and treatment planning. For providers who may be ill-equipped or reluctant to address S/R concerns, MHCs are available for consultation and collaboration. Further, MHCs help ID team members become more aware of and comfortable addressing the S/R domain when addressing MI by either providing training to help them recognize and address spiritual distress or seek support from MHCs in the resolution of S/R issues. Finally, MHCs can provide spiritual support to ID members facing their own personal and professional S/R struggles as they support those who have sustained a MI.

## Clinical perspectives

A MHC in the CAF, one of the co-authors (JSM) has been a key member of ID teams. Recently appointed Principal Chaplain for Mental Health by the Chaplain General, he has supported military members struggling with mental and spiritual health concerns including PTSD, MI and other OSI symptoms and facilitated their treatment. Referrals received from psychologists, social workers, psychiatrists, nurses or physicians typically focused on addressing grief, guilt and/or shame, or using S/R coping strategies to support recovery. Common interventions provided included bridging members to services and supports, facilitating S/R coping and grounding techniques, reconciling worldviews, resolving anger at a God-figure (not specific to any S/R perspective), and fostering reconciliation. Following is a brief discussion of ways in which MHCs contribute to healing in these areas.

### Bridging to services and supports

Chaplains are well-positioned to enhance member resilience and recognize distress, as well as act as a bridge to health and family services and supports. Embedded with the troops in theater and garrison, as well as in MH Clinics, Chaplains walk with service members during all stages of their military careers. In so doing, they provide support regarding prevention, promotion and recovery, and offer on-call front-line response for acute and chronic challenges. Their ongoing presence can be a reminder that others are available to extend non-judgmental care and of the value of S/R practices, resources and communities. They also can be facilitative of connection to other services. Contact with Chaplains has been found to diminish potential stigma associated with help-seeking and increase the likelihood that military personnel will be more receptive to interventions essential to recovery. The pre-established relationship between Chaplains and military members can be an invaluable asset in the process of recovery.

### Facilitating spiritual coping and grounding

MHCs can support members to effectively cope with MI and other OSI conditions, reduce stress and decrease arousal, and contend with the challenges of recovery by encouraging the use of S/R practices. Introduced early in treatment, S/R practices that facilitate grounding (e.g., meditation or contemplation) can become a source of comfort that then enables members to remain grounded throughout the course of treatment. Various studies show the impact of S/R grounding techniques on the brain and body in helping to reduce stress and decrease arousal. These practices can also help individuals to direct their attention to the “here and now,” grow in self-awareness, and reconnect with their body, mind, and spirit.

### Reconciling worldviews

Some military members have indicated that deployment changed or challenged their worldviews and beliefs. In this context, MHC interventions primarily center around exploring the person's S/R beliefs and values to identify what had previously been held as “sacred” and what has been lost or challenged since deployment. As an example, military members have had difficulties processing wartime encounters with child soldiers or casualties as it conflicts with a western worldview that holds children as sacred and innocent. Disparate representations of children (harmless vs. aggressors/threats) causes them to re-evaluate their perspective, beliefs and values. Such experiences can significantly impact a person's worldview and result in a redefinition of both self and self-in-relationship to others, the world and the sacred/Transcendent.

### Resolving anger at a god-figure

While military members may report having no precise S/R affiliation, they nonetheless often report feeling angry with a God-figure and being challenged by existential questions such as “Why is this possible?” “If God exists, why did God let this happen?” In some cases, operational experiences reinforce atheistic interpretations of events; in others, the experience can lead individuals to deepen their S/R beliefs. A deeper understanding of a psychosocial-S/R dilemma can be facilitated through MHC interventions. Provision of S/R support can help members formulate and integrate a new framework based on an evolving understanding of their relationship with a God-figure or what they consider sacred/Transcendent.

### Fostering reconciliation

Events occurring during deployment can also lead some military members to seek S/R interventions associated with forgiveness as a means of mending relationships with self, others and/or the sacred/Transcendent. Interventions related to reconciliation employed by MHCs center on distress, guilt and/or shame and are personalized according to S/R needs and resources. In the process of seeking forgiveness, valuing self in relation to others and a God-figure can be challenging for some individuals. Liaising with local clergy or spiritual leaders regarding the sacrament of reconciliation or other rituals can be supportive; in other cases, dialogue with a benevolent authority has been central to the acknowledgment of guilt and extension of forgiveness.

The above clinical perspectives shed light on some of the contributions of MHCs in supporting the recovery of military members dealing with OSIs, PTSD, and MI. Addressing divergent worldviews, and anger at a God-figure, drawing on S/R resources including coping and grounding techniques, and supporting reconciliation can address fundamental issues, help facilitate recovery and enable military members and veterans to be more receptive to and ready for other treatment modalities available through the ID team.

## Discussion

The success of integrating MHCs into CAF Mental Health Clinics, which has been affirmed by ID teams and service members, offers evidence of the therapeutic value they offer in the treatment of MI. Most significantly, MHCs on ID teams ensure that: (1) spiritually-integrated holistic person-centered care is delivered, (2) S/R interventions are integrate into treatment for MI, and (3) capacity-building of ID teams to address this domain in relation to MI is facilitated.

### Ensuring holistic person-centered care

Addressing the needs of military members from a holistic, BPSS perspective is essential to healing from MI. MHCs on ID teams are well-positioned to encourage military members to reflect upon themselves and their spiritual processes (e.g., struggles, questions and wounds), and where appropriate, use various S/R interventions (practices) to support healing and reintegration post-MI. Considering MI as merely a collection of psychological symptoms to the negation of S/R components may impede recovery. Nash et al. (15) argue that the use of spiritual language and practices should be encouraged for the treatment of MI given its underlying spiritual dimension. Equally, developing, strengthening, or restoring the human spirit, providing comfort through S/R means, and facilitating transition through and closure for experiences and chapters of life have long been key contributions of S/R.

### Integrating S/R interventions

Pertinent to the treatment of MI is the ability of MHCs to integrate S/R processes and practices. As illustrated in the literature and observed clinically, MHCs are uniquely qualified to engage in dialogue regarding the spiritual domain, assess for S/R strengths and distress, and integrate S/R processes and practices in the context of the overall ID treatment plan. Successful treatment for MI requires recognizing MIEs and supporting individuals as they seek to re-integrate their core self, reframe their worldview, and re-establish relationships with themselves, others, and the sacred or a God-figure. Addressing a person's S/R values, beliefs, needs and resources in the course of treatment may alleviate pathology, enable individuals to attain optimal mental, physical, spiritual, and social functioning (43), and increase their receptivity to other supports and services. Moreover, spiritual well-being activities, specifically S/R coping (e.g., meaning-making, support, ritual, practice, meditation), can offer a distinctive benefit over and above the effects of secular methods of coping (13, 45).

### Facilitating S/R capacity-building

Addressing the spiritual dimension of MI is not common-place for many mental health clinicians. Finlay (46) notes that there currently exists a dearth of clinicians capable of appropriately addressing the S/R components of MI (46). As some ID team members may feel ill-equipped to address S/R aspects of MI, MHCs can assist them to: (1) develop a therapeutic alliance that is sensitive to the S/R domain; (2) assess and identify S/R concerns; (3) include spirituality in treatment planning; (4) implement spiritually-integrated psychological interventions; (5) monitor and evaluate overall treatment progress and outcomes of S/R goals; (6) prevent compassion fatigue and work within practice guidelines; and (7) be aware of attitudes regarding S/R that they may bring into their practice (47, 48). Plante (47) encourages healthcare providers integrating S/R into clinical care to be aware of biases, consider S/R like any other type of diversity, take advantage of available resources, and consult colleagues including Chaplains and S/R leaders (47).

## Conclusion

This perspective paper has explored the concept of MI, the role of Chaplains in its identification and treatment, and ways in which S/R interventions delivered by MHCs can be facilitative of recovery. When MI occurs, all dimensions of the person need to be addressed including underlying spiritual concerns. From a spiritual perspective, pursuit of healing and wholeness following exposure to an MIE often involves facing existential and S/R questions, finding healing for wounds in one's deep core, and mending fractured relationships with self, others and the sacred/Transcendent. At times, spiritual blocks need to be overcome and worldviews, beliefs and values reconciled in order for members to be able to more fully benefit from other forms of therapy. While all members of the ID team may address the spiritual domain to some degree, Chaplains, and more particularly MHCs, are well-positioned to help military members face and make sense of the spiritual aspects of MIEs. It is the opinion of the authors that, given the empirical evidence, feedback from ID team members, and clinical experience, that MHCs should be fully integrated as part of any ID team working with military personnel and veterans who experience mental and spiritual health concerns including MI.

## Author contributions

LS-M, J-SM, and SB-P participated in the conceptualization and writing of this article and approved the final version. In doing so, all authors agree to be accountable for the content of the work.

### Conflict of interest statement

The authors declare that the research was conducted in the absence of any commercial or financial relationships that could be construed as a potential conflict of interest.
